# Signal Transduction of Platelet-Induced Liver Regeneration and Decrease of Liver Fibrosis

**DOI:** 10.3390/ijms15045412

**Published:** 2014-03-28

**Authors:** Soichiro Murata, Takehito Maruyama, Takeshi Nowatari, Kazuhiro Takahashi, Nobuhiro Ohkohchi

**Affiliations:** Department of Surgery, Faculty of Medicine, University of Tsukuba, 1-1-1 Tennodai, Tsukuba, Ibaraki 305-8575, Japan; E-Mails: soichiro@md.tsukuba.ac.jp (S.M.); s1030518@u.tsukuba.ac.jp (T.M.); s1130448@u.tsukuba.ac.jp (T.N.); kazu1123@hh.iij4u.or.jp (K.T.)

**Keywords:** platelet, STAT3, Akt, ERK1/2, S1P, adenosine, cyclic AMP

## Abstract

Platelets contain three types of granules: alpha granules, dense granules, and lysosomal granules. Each granule contains various growth factors, cytokines, and other physiological substances. Platelets trigger many kinds of biological responses, such as hemostasis, wound healing, and tissue regeneration. This review presents experimental evidence of platelets in accelerating liver regeneration and improving liver fibrosis. The regenerative effect of liver by platelets consists of three mechanisms; *i.e.*, the direct effect on hepatocytes, the cooperative effect with liver sinusoidal endothelial cells, and the collaborative effect with Kupffer cells. Many signal transduction pathways are involved in hepatocyte proliferation. One is activation of Akt and extracellular signal-regulated kinase (ERK)1/2, which are derived from direct stimulation from growth factors in platelets. The other is signal transducer and activator of transcription-3 (STAT3) activation by interleukin (IL)-6 derived from liver sinusoidal endothelial cells and Kupffer cells, which are stimulated by contact with platelets during liver regeneration. Platelets also improve liver fibrosis in rodent models by inactivating hepatic stellate cells to decrease collagen production. The level of intracellular cyclic adenosine monophosphate (cyclic AMP) is increased by adenosine through its receptors on hepatic stellate cells, resulting in inactivation of these cells. Adenosine is produced by the degradation of adenine nucleotides such as adenosine diphosphate (ADP) and adenosine tri-phosphate (ATP), which are stored in abundance within the dense granules of platelets.

## 1. Introduction: Cirrhosis

Cirrhosis is a serious and life-threatening major health problem worldwide. It is an advanced form of hepatic fibrosis in response to chronic liver injury [1]. Up to 64% of patients with cirrhosis suffer thrombocytopenia [2–5]. Patients with thrombocytopenia sometimes cannot receive antiviral therapy with sufficient doses of interferon for hepatitis virus or curative surgery for hepatocellular carcinoma. When standard therapy has failed to control cirrhosis, liver transplantation is the only effective therapy [6]. Unfortunately, liver transplantation is associated with donor shortage, surgical complications, organ rejection, and high cost [7–11]. Patients are often required to wait for many years for liver transplantation because of the shortage of donor organs, and some of them die while waiting. Therefore, alternative treatments are required to treat patients with cirrhosis. Treatment of cirrhosis consists of anti-inflammation, liver regeneration, and improvement of fibrosis. Each of these present very challenging problems in the clinical settings and no clear solutions have been found yet.

## 2. Platelets

Platelets are derived from megakaryocytes (MKs). Megakaryocytes are derived from multipotent hematopoietic stem cells toward MK progenitors. Mature MK produces platelets by cytoplasmic fragmentation occurring through a dynamic and regulated process, called proplatelet formation, and consisting of long pseudopodial elongations that break in the blood flow [12]. Platelets are discoid and have anucleate structures that contain a large number of secretary granules [13]. Three types of secretary granules are recognized: alpha granules, dense granules, and lysosomal granules. Each granule contains secretory substances, such as platelet-derived growth factor (PDGF), insulin-like growth factor-1 (IGF-1), hepatocyte growth factor (HGF), vascular endothelial growth factor (VEGF), serotonin, adenosine diphosphate (ADP), adenosine tri-phosphate (ATP), epidermal growth factor (EGF), and transforming growth factor-β (TGF-β), *etc*. [13–16]. Platelets are activated by various types of stimulation and release active substances from the granules [13–19]. Several positive effects have been reported, such as hemostasis [17], wound healing [20–23], and tissue regeneration [24–29]. On the other hand, there are some negative effects of platelet degranulation, such as inflammation [30], malignancy [31,32], and immune response [33–36]. Platelets are reported to accumulate in the liver under pathological conditions such as ischemia/reperfusion [37–40], liver cirrhosis [41], cholestasis [42], viral hepatitis [43], and the residual liver after hepatectomy [44].

## 3. Platelets and Liver Regeneration

The proliferative effect of platelets in liver regeneration was first reported in 2006 by Lesurtel *et al.*, who suggested platelet serotonin is important for liver regeneration [45]. Our first study focused on liver regeneration under thrombocytotic conditions induced by thrombopoietin (TPO) [44]. TPO is a growth factor that regulates the development of MK and platelet production [46]. c-Mpl is a TPO receptor, and several novel agents that stimulate human c-Mpl and increase platelet levels, such as eltrombopag and romiplostim, are used for the treatment of chronic immune thrombocytopenia [46,47]. We used 0.5 μg of pegylated recombinant human MK growth and development factor (PEG-rHuMGDF) donated by Kirin Brewery Co. (Takasaki, Japan) as TPO [44]. TPO was administered intraperitoneally five days before hepatectomy, which increases the peripheral platelet count two to three-fold over the pre-administration state in mice [44]. Thrombocytotic conditions promote liver regeneration including the liver/total body weight ratio, hepatocyte Ki-67 labeling index and mitotic index [44,48], and improve the survival rates after 90% hepatectomy of mice [49]. A significant increase in the hepatic concentrations of HGF and IGF-1 and early and strong phosphorylation of Akt and signal transducer and activator of transcription-3 (STAT3) are induced under thrombocytotic conditions [44,48]. Platelet-rich plasma transfusion was performed after partial hepatectomy, and liver regeneration was accelerated [50]. These findings indicate that exogenous platelets also have an impact on liver regeneration through the early initiation of hepatocyte cell cycles after hepatectomy. The effect of TPO administration on liver regeneration under cirrhotic liver and its anti-fibrosis effects were evaluated after partial hepatectomy [51]. TPO increased peripheral platelets and promoted liver regeneration, including increasing the hepatocyte-proliferating cell nuclear antigen (PCNA) labeling index and mitotic index in the cirrhotic liver, and improved fibrosis in the peri-portal regions [51]. The proliferative effect on acute liver damage after hepatectomy by increasing platelets in a pig model was reported [52]. Cholestasis, ballooning, and necrosis in the liver are decreased under thrombocytotic conditions and serum aspartate amino transferase and alkaline phosphatase levels are low after extended hepatectomy [52]. Transmission microscopy revealed that the structure of the endothelial lining is well preserved in comparison with the structure observed with a normal platelet count [52]. The increased number of platelets protects the sinusoidal lining and prevents acute liver damage after extended hepatectomy. Platelets also protect against Fas-mediated apoptosis of hepatocytes in murine acute hepatitis model induced by anti-Fas antibody [53]. Platelets induce immediate activation of the Akt pathway, followed by an increase of B-cell lymphoma-extra large (BCl-xL) and a decrease of cleaved caspase-3 in hepatocytes [53].

## 4. Signal Transduction of Liver Regeneration by Platelets

**Figure 1.**
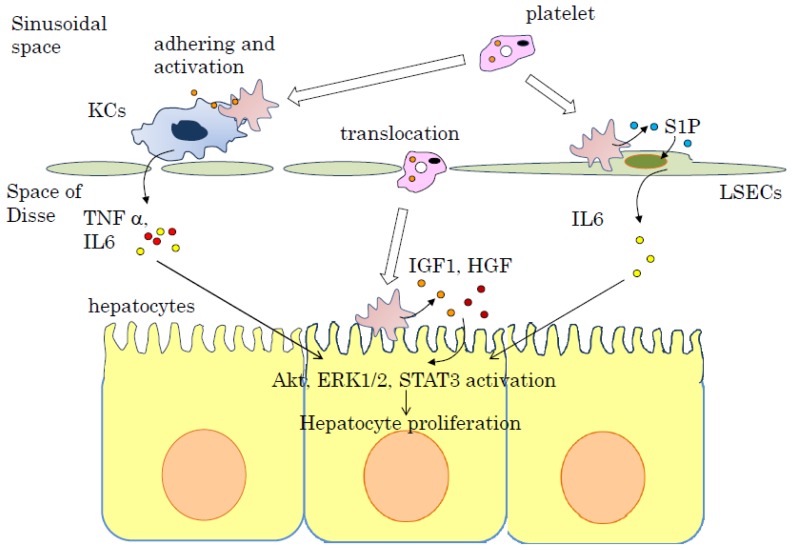
Liver regeneration promoted by platelets. Platelets accumulate in the liver immediately after hepatectomy. Platelets translocate from the sinusoidal space to the space of Disse and release growth factors such as insulin-like growth factor-1 (IGF-1) and hepatocyte growth factor (HGF) through direct contact with hepatocytes, which subsequently induce initiation of hepatocyte mitosis; the direct contact between platelets and liver sinusoidal endothelial cells (LSECs) triggers the release of sphingosine 1-phosphate (S1P) from platelets, which leads to excretion of interleukin-6 (IL6) from LSECs. IL6 from LSECs promotes proliferation of hepatocytes; and Kupffer cells (KCs) and platelet interaction activated KCs after hepatectomy. Activated KCs release tumor necrosis factor-α (TNFα) and IL6. IGF-1 and HGF activates Akt and ERK1/2 in the hepatocytes. IL6 stimulates STA3 activation. These signal transduction molecules proliferate hepatocytes.

*In vivo*: platelets accumulate in the liver immediately after hepatectomy. Under normal conditions, liver sinusoidal cavities show intact endothelial lining consisting of liver endothelial cells with flattened processes perforated by small pores [54]. These small pores are reported to quickly enlarge within 10 min after hepatectomy [54,55]. In response to lipopolysaccharide administration, interleukin-1 or tumor necrosis factor, platelets accumulate in the liver sinusoidal space within a few minutes, stimulated by a different mechanism of aggregation, and large numbers of platelets are found in the space of Disse and even inside some hepatocytes [56–58]. In our study, platelet accumulation in the liver was observed in thrombocytotic groups in the early period after hepatectomy. In addition, platelets translocated into the space of Disse and had direct contact with hepatocytes 5 min after hepatectomy in the thrombocytosis group, which could be observed by transmission electron microscopy [44]. Platelets are translocated from the liver sinusoids to the space of Disse, and growth factors such as HGF, IGF-1, and VEGF could be released through direct contact between platelets and hepatocytes (Figure 1). These soluble mediators lead to hepatocyte proliferation. Human platelets are reported to have a very limited amount of HGF [59], thus IGF-1 is considered to be the most important mediator for liver regeneration in human platelets. The Akt and ERK1/2 pathways in whole liver extracts are activated immediately after hepatectomy in thrombocytotic mice, compared with thrombocytopenic mice [44].

*In vitro*: the signal transduction of hepatocytes, which is activated by platelets, was analyzed. The phosphorylation of Akt and extracellular signal-regulated kinase (ERK)1/2 were analyzed in murine immortalized hepatocyte TLR2 and primary cultured murine hepatocytes stimulated by platelets [60]. The Akt pathway and ERK1/2 pathway are activated within 10 min after adding platelets into the culture medium of hepatocyte *in vitro* [60]. The Akt pathway, which is activated by growth factors, is known as a survival signaling pathway [61,62]. The ERK1/2 pathway, which is also activated by growth factors, is involved in growth and differentiation [63].

Liver sinusoidal endothelial cells (LSECs) enable contact between circulating blood and hepatocytes and help to exchange various soluble macromolecules and nano-particles, such as hyaluronic acid and lipoproteins [54]. LSECs are known to produce growth factors, such as HGF and VEGF and pro-inflammatory cytokine interleukin (IL)-6 and promote liver regeneration after hepatectomy. Elevation of IL-6 concentration after hepatectomy activates the acute phase of protein synthesis by hepatocytes [64]. IL-6 binds to the receptor on hepatocytes, which leads to phosphorylate STAT3 monomers. The relationship between platelets and LSECs is addressed in ischemia/reperfusion models [65,66], but there have been very few previous studies that focused on the relationship between platelets and LSECs during liver regeneration before our study [67]. The role of platelets in liver regeneration in relation to LSECs was evaluated by co-culturing chamber systems *in vitro*. This study revealed that direct contact between platelets and LSECs induce IL-6 release from LSECs, and IL-6 derived from LSECs accelerates hepatocyte proliferation. In addition, sphingosine-1-phosphate (S1P) in platelets plays an important role in IL-6 secretion [67] (Figure 1). S1P is a lipid mediator that regulates many kinds of biological processes including proliferation, migration, and cytoskeletal reorganization [67]. S1P is excreted from activated platelets and interacts with endothelial cells under the conditions of thrombosis, angiogenesis, atherosclerosis, and liver regeneration [67–69]. Zheng *et al.* revealed that S1P protects LSECs from alcohol-induced apoptosis via activation of eNOS [70]. Isabel Fernández-Pisonero *et al.* revealed that S1P combined with lipopolysaccharide (LPS) activates human umbilical endothelial cells (HUVECs), famous endothelial cells, via NF-κB, ERK1/2, and p38 and activated HUVECs secrete IL-6 [71].

Kupffer cells play a role in the liver as resident macrophages that protect the liver from bacteria, endotoxins, and microbial debris derived from the gastrointestinal tract [72]. Kupffer cells produce important cytokines that enable hepatocyte proliferation after hepatectomy [73]. One of the most important events after hepatectomy is an increase in the plasma levels of tumor necrosis factor (TNF)-α. An experiment using an antibody against TNF-α demonstrated a significant reduction of hepatocyte proliferation [74], and mice lacking the TNF-α receptor showed severe impairment in liver regeneration after hepatectomy [75,76]. The activation of the TNF-α receptor increases hepatic expression of the NF-κB in both hepatocytes and non-parenchymal cells, and is followed by production and release of IL-6 from Kupffer cells [77]. Kupffer cells are considered to be the most important source of both TNF-α and IL-6. Kupffer cell-depleted mice fail to increase TNF-α and IL-6 levels that are equivalent to the level in mice with Kupffer cells after hepatectomy [78]. The collaborative effect of platelets with Kupffer cells on liver regeneration is thought to occur after hepatectomy, when activated Kupffer cells induce accumulation and activation of platelets in the liver, and the functions of Kupffer cells are enhanced by the accumulated platelets. Liver regeneration is promoted by the direct effect of growth factors released from platelets and by the paracrine effect of Kupffer cells enhanced by the platelets [79] (Figure 1).

## 5. Effect of Platelets and Thrombopoietin Receptor Agonist in Liver Cirrhosis

As mentioned previously, several novel agents that stimulate human c-Mpl and increase platelet levels, such as eltrombopag and romiplostim, are used for the treatment of chronic immune thrombocytopenia [46,47]. These agents are currently in development for the treatment of thrombocytopenia in patients with chronic liver disease and liver cirrhosis [80–82]. The ability to increase platelet count could facilitate the use of interferon-based antiviral therapy and other treatments for liver disease [3,83].

It was reported that the increment of platelets induced by TPO administration could improve liver fibrosis in experimental studies with rodents [51,84]. Dimetylnitrosamine was administered three times a week for three weeks to induce liver fibrosis in rats. Five days after administrating TPO intravenously, 70% hepatectomy was performed and liver fibrosis was compared 24 h after hepatectomy. The increase of platelets inhibited the activation of hepatic stellate cell (HSC) and reduced the fibrotic area of the cirrhotic liver, and these effects were diminished by administration of antiplatelet serum [51]. Carbon tetrachloride (CCL4) was administered twice a week for eight weeks to induce liver fibrosis in mice. TPO was administered intraperitoneally once a week from five to eight weeks during the experiment [84]. By administering TPO, liver fibrosis was decreased [84]. Although the precise mechanisms between the increment of platelets and the liver anti-fibrotic effect are still unclear, one reason may be that platelets enhanced the expression of HGF by about 14% [51], whereas the matrix metalloproteinase 9 (MMP9) was enhanced by about three times, thereby stimulating fibrolysis, and decreased pro-fibrotic growth factor TGF-β [84]. MMPs such as MMP-8, MMP-9, and MMP-13 possess the ability to degrade the extracellular matrix by breakdown of collagen type I [85–87]. MMP-9 may indirectly contribute to fibrolysis by accelerating HSC apoptosis [88]. In murine bile duct ligation model, thrombocytopenia exacerbates liver fibrosis, and platelets have anti fibrotic role in suppressing type I collagen expression via the HGF–Met signaling pathway [89]. Recently, Takahashi *et al.* reported that transfused human platelets improved liver fibrosis of severe combined immune deficiency (SCID) mice induced by CCL4 [90]. An increase of murine HGF and a decrease of TGF-β were observed in the liver [90].

Based on these animal experiments, clinical trial was performed. Maruyama *et al*. recently reported the clinical trial to investigate whether platelet transfusion improves liver function in patients with chronic liver disease and cirrhosis (Child-Pugh class A or B), who all presented thrombocytopenia (platelet counts between 50,000 and 100,000/μL). The subjects received 10 units of platelet concentrate once a week for 12 weeks. One and three months after the last transfusion, significant improvement of serum albumin was observed. Serum cholinesterase improved for nine months after the last transfusion. Serum hyaluronic acid represents liver fibrosis, and that showed a tendency toward improvement after the last transfusion [91].

## 6. Signal Transduction of Liver Fibrolysis Induced by Platelets and Thrombopoietin (TPO)

**Figure 2.**
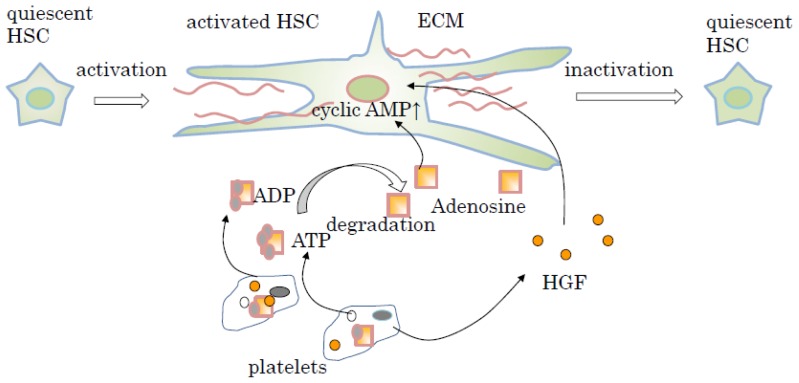
Scheme showing the functions of platelets in the suppression of liver fibrosis. Under chronic viral infection, quiescent hepatic stellate cells (HSCs) become activated and produce a large amount of extracellular matrix (ECM) and participate in the progression of liver fibrosis. After the treatment to increase the platelet count, including administration of thrombopoietin (TPO) and platelet transfusion, platelets come in contact with HSCs and release adenine nucleotides such as adenosine 5′-diphosphate (ADP) and adenosine 5′-triphosphate (ATP). These adenine nucleotides subsequently lead to the production of adenosine through the degradation by HSCs. Adenosine is incorporated to HSC via the adenosine receptor and increase cyclic AMP in the HSCs. Increased cyclic AMP plays an important role in the inactivation of HSCs. Platelets also contribute to the expression of hepatocyte growth factor (HGF) in the liver. Activated HSCs are inactivated by adenosine or HGF to reduce the production of ECM.

In the long-term natural history of chronic liver injury, such as viral infection, alcohol, and non-alcoholic steatohepatitis, liver fibrosis occurs. Liver fibrosis is known to be part of a dynamic process of continuous extracellular matrix (ECM) remodeling, which leads to the excessive accumulation of several extracellular proteins, proteoglycans, and carbohydrates [92]. Among the cellular populations in the liver, HSCs are reported to have the most involvement in liver fibrosis through the production of large amounts of ECM and the secretion of TGF-β, which appears to be a key mediator of liver fibrosis [93–95]. In the response to liver injury, HSCs are activated to convert from vitamin A storing star-like cells into contractile myofibroblastic cells [92]. Recently, Ikeda *et al.* reported that human platelets contributed to the suppression of HSC activation and reduction of type I collagen production *in vitro* [96]. The level of intracellular cyclic adenosine monophosphate (cyclic AMP) is increased by adenosine through its receptors on HSCs, and intracellular cyclic AMP is related to the inactivation of HSCs (Figure 2). Large amounts of adenosine around HSCs are produced by the degradation of adenine nucleotides such as ADP and ATP, which are stored in abundance within the dense granules of platelets. It is possible to say that activated HSCs are inactivated by adenosine and have a decreased ability to produce TGF-β and secrete ECM [96] (Figure 2). In rodents, platelet-derived HGF suppresses TGF-β and type I collagen gene expression in cultured HSCs [89] (Figure 2). These findings indicate that platelets suppress liver fibrosis by inactivating HSC.

## 7. Conclusions

This review discussed previous evidence of platelets promoting liver regeneration and improving liver fibrosis. There are three different mechanisms of liver regeneration induced by platelets: (i) direct effect on hepatocytes; (ii) a cooperative effect with LSECs; and (iii) a cooperation with Kupffer cells. There is significant evidence that platelets play a role in improving fibrosis. ATP and ADP inside platelets are degraded by HSCs and adenosine is incorporated into HSCs. Cyclic AMP is increased by adenosine and HSCs become inactivated by cyclic AMP. Therefore, platelet therapy, *i.e.*, platelet transfusion and TPO receptor agonist administration would open a new avenue to develop novel strategies for the treatment of liver diseases for which there is currently no effective treatment except transplantation.
